# Isolated Hydatid Cyst in a Single Moiety of an Incomplete Duplex Kidney

**Published:** 2015-01-01

**Authors:** Vinod Priyadarshi, Shwetank Mishra, Malay Kumar Bera, Dilip Kumar Pal

**Affiliations:** Department of Urology, Institute of Postgraduate Medical Education and Research, Kolkata, India.

**Keywords:** Hydatid cyst, Echinococcosis, Duplex kidney

## Abstract

Isolated hydatid cyst of kidney is very rare. Hydatid cyst of a duplex renal system is even more rare. We report a 13-year old girl with duplex system of right kidney with isolated hydatid cyst in upper moiety. Right nephrectomy was done to cure the condition.

## INTRODUCTION

Kidney involvement in echinococcosis is extremely rare. Primary involvement of the kidney without the involvement of the liver and lungs is even rarer.[1] Though, a duplex collecting system is one of the most common congenital renal tract abnormality, it is seen in only 0.7% of the normal adult population and in 2-4% of patients investigated for urinary tract symptoms.[2,3] There are only a few case reports of hydatid cyst in an anomalous kidney and most were in Horseshoe kidneys.[4,5] We present a case of primary renal hydatid cyst in upper moiety of right duplex kidney.

## CASE REPORT

A 13-year old female presented with continuous dull pain localized to the right flank region for three months. She had no urinary complaints. General examination was unremarkable. Abdominal examination revealed a 10 cms x 10 cms lump in the right lumbar region which was bimanually palpable. Her routine blood and urine investigations were normal with no eosinophilia, and had normal renal function tests. X-ray chest was normal. Ultrasonography whole abdomen suggested a heterogeneous cyst of size 13 cms x 10 cms with multiple daughter cysts and septation in the right lobe of liver with non-visualisation of right kidney. Contrast Enhanced Computed Tomography (CECT) showed a large multiloculated cyst of size 11.5cms x 10.5cms x 15.5cms with internal septations arising from the upper pole of right kidney, with typical cysts within cyst appearance and thinned out peripheral parenchyma. Liver was pushed upwards and medially, which otherwise was normal (Fig.1). ELISA test for echinococcus antibodies was positive. A course of albendazole was given for one month and subsequently patient was planned for surgery.

**Figure:**
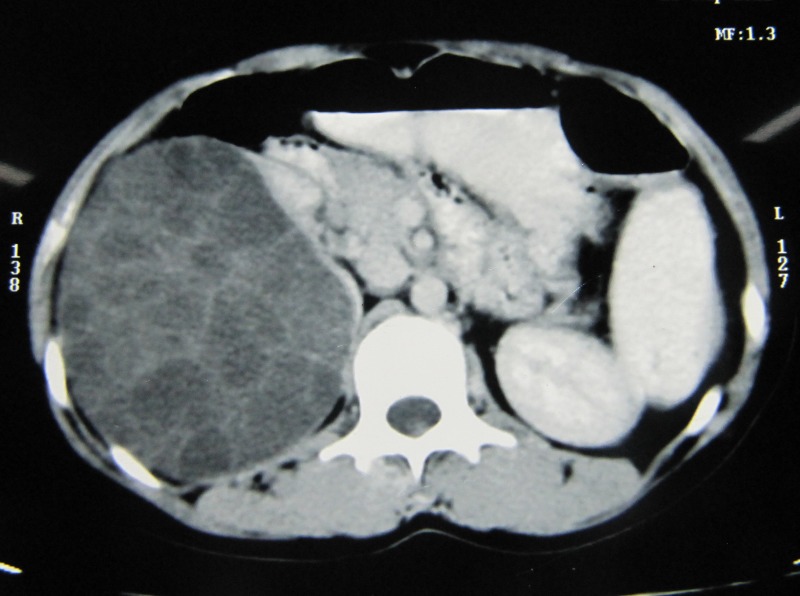
Figure 1:CECT showing multiloculated cystic lesion in the upper pole of right kidney with typical cysts within cyst appearance and thinned out peripheral parenchyma.

On exploration, there was a large hydatid cyst in the upper moiety of right incomplete duplex kidney, compressing the lower moiety into thin rim of parenchyma with two separate ureters that were united caudally. Heminephrectomy was not feasible, so a simple nephrectomy was done. Cut specimen of the right kidney had two moieties with two ureters. The upper moiety was turned into a bag of cysts while lower moiety had compressed thinned out renal parenchyma with no evidence of any cystic disease (Fig. 2,3). The histopathological examination of the cyst was consistent with the diagnosis of hydatid disease of kidney. Albendazole was continued for eight weeks after surgery. USG abdomen after 6 months suggested no recurrence.

**Figure:**
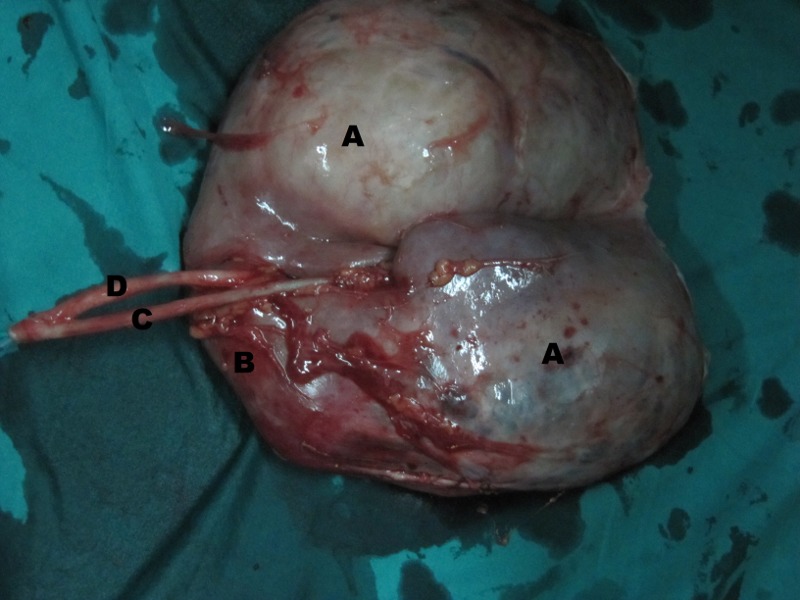
Figure 2:Gross cut open specimen (outer suface) showing A). Cystic upper moiety, B).Compressed lower moiety, C). Ureter from upper moiety,and D). Ureter from lower moiety.

**Figure:**
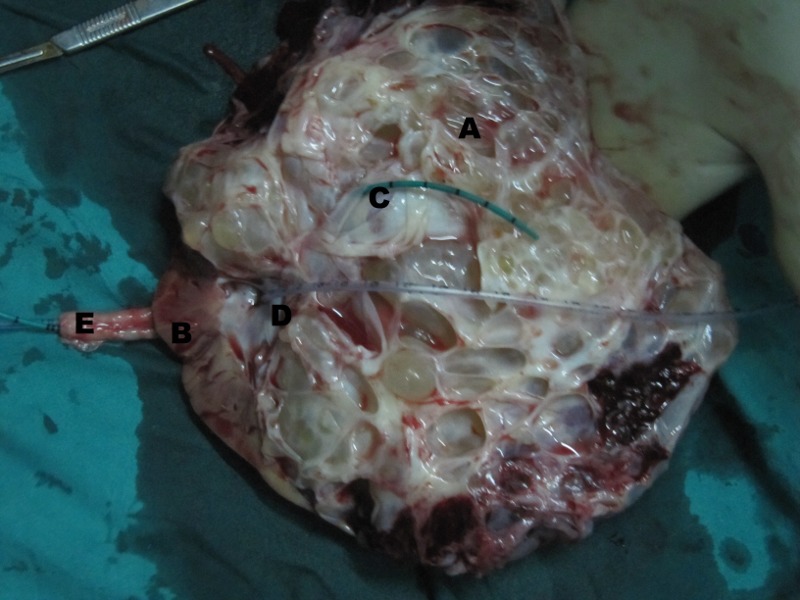
Figure 3:Gross cut open specimen (inner surface) showing A). Hydatid Cyst in upper moiety, B).Compressed lower moiety, C).Opening of upper pelvicalyceal system with a ureteric catheter within, D).Opening of lower moiety pelvicalyceal system with an infant feeding tube within, and E). Two ureters.

## DISCUSSION

It is postulated that the renal hydatid cyst form when the echinococcous larvae are lodged into the kidneys through retroperitoneal lymphatics while passing through portal system to the liver or through systemic circulation after passing liver and lungs.[6] The hydatid cyst of the kidney is considered closed if all three layers of the cyst i.e. pericyst, ectocyst and endocyst are intact.[1] When the cyst is no longer protected by the third layer, i.e pericyst or by the lining of the collecting system, it is considered to be an exposed cyst. If all the three layers of the cyst have been ruptured, resulting in free communication with the calyces and pelvis, it is called an open or communicating cyst.[7] Cystic rupture into the collecting system, causing hydatiduria is pathognomonic of renal hydatidosis, though it is usually microscopic and is seen in only 10-20% of renal hydatidosis.[1,7]

Renal hydatid cysts are usually asymptomatic or may present with chronic dull pain or lower backache due to enlarging cyst.[6] Rarely they may present with colicky pain due to hydatiduria and chronic renal failure when bilateral kidneys are involved.[6] A thin rim of calcification delineating a cyst is suggestive of an echinococcal cyst. Ultrasonography helps in the diagnosis of hydatid cysts when the daughter cysts and hydatid sand are demonstrated. On changing the patient's posture under real time, there is shifting of hydatid sand, which may give rise to the "falling snowflake pattern".[7] The mainstay of diagnosis is by advanced radiological techniques like CT scan and magnetic resonance imaging (MRI).[1,8] CECT has an accuracy of 98% to demonstrate the daughter cysts. The CT scan usually demonstrates an expansile, hypo-attenuating tumor with a well-defined wall and daughter cysts within the parent cyst.[8,9] MRI usually reveals a solitary, high-signal-intensity mass consisting of multiple thin-walled lesions and outlined by a thick, hypointense rim. The high signal intensity is due to the characteristic high fluid content of the mass. The small peripheral cysts are usually hypointense relative to the central component. The MRI shows the cysts adequately, but MRI offers no real advantage over CT scan.[1,8,9]

Surgery is the treatment of choice in cases of renal hydatidcyst.[6] Kidney sparing hydatid cyst removal (cystectomy with pericystectomy), is possible in most (75%) cases. Nephrectomy is considered only if the kidney is destroyed by the cyst. Both open and laparoscopic techniques have been described. There is risk of cyst rupture and dissemination during laparoscopy.[6] During kidney-sparing surgery scolicidal solutions such as hypertonic saline should be used. Pre- and postoperative course of albendazole is recommended to sterilize the cyst, decrease the chance of anaphylaxis and decrease the tension in the cyst wall thus reducing the risk of spillage during surgery and recurrence.

## Footnotes

**Source of Support:** Nil

**Conflict of Interest:** None declared

